# Study on the relationship and present situation between standardized training nurses of spiritual climate and psychological detachment in Sichuan Province, China

**DOI:** 10.3389/fmed.2025.1716555

**Published:** 2026-01-09

**Authors:** Juan Zhao, Rong Huang, Yanlin Wei, Xu Ran, Zhen Yang, Chenxi Tang

**Affiliations:** 1 Beijing Anzhen Nanchong Hospital, Capital Medical University & Nanchong Central Hospital, Nanchong, Sichuan, China; 2 The Second Clinical Medical College of North Sichuan Medical College, Nanchong, China

**Keywords:** present situation, psychological detachment, relationship, spiritual climate, standardized training nurses

## Abstract

**Objective:**

To investigate the current situation of nurses’ perception of spiritual climate and psychological detachment in standardized training (regulation training) in Sichuan Province, China, and to explore the relationship and influencing factors between them.

**Methods:**

From August to September 2024, a convenience sampling method was adopted to conduct an electronic questionnaire survey among 716 standardized training nurses in 8 tertiary hospitals in Sichuan Province. The survey tools included a general information questionnaire, the Chinese version of the spiritual climate brief scale and the Chinese version of the psychological detachment scale. SPSS 26.0 was used for descriptive analysis, nonparametric test, Spearman correlation analysis and generalized linear model analysis.

**Results:**

The median score of spiritual climates in standardized training nurses was 16 (full score 20), and the median score of psychological detachment was 11 (full score 20), both of which were at a medium level. Spiritual climate was significantly positively affected by age (higher for those ≤20 years old), How much you love your career (the more you love it, the higher), colleague relationship (higher for those who were harmonious) and personality (higher for those who were introverted); Psychological separation was significantly related to height (Those who were >165 cm scored higher) and formal work experience before training (Those with work experience scored higher). There was a weak positive correlation between spiritual climate and psychological detachment (*r* = 0.081, *p* = 0.029).

**Conclusion:**

The spiritual climate and psychological detachment levels among nurses in Sichuan Province’s standardized training programs require improvement, with weak correlations between the spiritual climate and psychological detachment. Key improvement priorities include enhancing departmental humanistic environments (particularly for those with low professional identity) and strengthening stress management along with work-life balance training (especially for inexperienced staff). Future research should delve deeper into the mechanisms linking height to psychological detachment.

## 1 Introduction

Standardized training (hereinafter referred to as standardized training) refers to the systematic clinical training that nursing staff receive after completing basic studies, which is an important way to cultivate clinical nursing talent echelon (1, 2). Since the implementation of the national Health Commission (3) training measures in 2016, more and more nurses have chosen to undergo systematic standardized training before formal employment. Nurses in training are in an unstable working environment for a long time, facing unfamiliar interpersonal relationships and high pressure work, which can easily lead to anxiety, depression, stress, frustration and other negative emotions (4). Spiritual climate (SC) is a common understanding among employees towards spirituality and the par taking of the shared spirituality of the workplace (5). A positive spiritual climate could make nurses productive, perform well at work and become emotionally intelligent, committed and satisfied at work (6). Psychological detachment refers to the ability of an individual to completely detach from work-related affairs during non-work time (7). In May 2021, foreign scholars (8) conducted a survey involving 1,111 South Korean nurses to explore the relationships between nursing teamwork, psychological detachment, and job burnout. The study revealed that nursing teamwork enables nurses to distance themselves from work during non-working hours, thereby enhancing their psychological detachment. Note that nursing teamwork is closely associated with the workplace spiritual climate. Effort-Recovery Theory (ERT) holds that individuals’ mental effort in work will consume mental resources, and if there is no effective recovery, it will easily lead to burnout and emotional exhaustion. “psychological detachment” is the key link of “restoration process,” which helps individuals to fully recover their physical and psychological resources (9). The core of the Effort-Recuperation Theory is the closed loop of “effort consumes resources—recuperation resources are released.” The spiritual climate is considered a positive external work resource, which can improve the work state and may indirectly influence the psychological detachment through the promotion of the work state. We discuss whether the spiritual climate can be an “external key variable” to influence the formation of this closed loop. Current research in China predominantly focuses on psychological detachment among patients and their caregivers, with limited attention paid to the mental health status of nurses in standardized training programs. This study investigates the spiritual climate and psychological detachment among these trainees, exploring their interconnections to provide evidence-based strategies for addressing mental health challenges in nursing education.

## 2 Objects and methods

### 2.1 Study subjects

From August to September 2024, 716 standardized training nurses from 8 tertiary hospitals in Sichuan Province were selected via convenience sampling. Inclusion criteria: ① Nurses with valid nursing practice certificates; ② Those actively participating in Sichuan Province’s standardized nurse training; ③ Individuals who voluntarily consented to the study. Exclusion criteria: Nurses absent during the survey period (e.g., on maternity leave, sick leave, or in the extended training phase). Ethics and recruitment: This study was approved by the Ethics Committee of Beijing Anzhen Nanchong Hospital, Capital Medical University & Nanchong Central Hospital (Approval No.: 2024-145). With the nursing department’s approval, participants were recruited through WeChat groups for standardized training nurses, where the study objectives and requirements were fully explained. Incentives: No monetary or material incentives were provided to participants. Data quality control: Participants completed questionnaires independently; duplicate submissions were prevented by limiting one response per mobile phone number/IP address. All participants provided informed consent, and their personal information was kept confidential.

### 2.2 Calculation of sample size

This study’s general data encompasses 17 variables, with the spiritual climate section containing 4 items and the psychological detachment section including 4 items, totaling 25 variables. Cross-sectional studies typically require a sample size of 5–10 times the number of independent variables, plus 20% invalid-response allowance, resulting in a calculated sample size of 300 cases. To increase the representativeness and accuracy of the samples, our study surveyed 729 registered nurses undergoing standardized training in Sichuan Province. To improve the representativeness of the sample, we selected two hospitals from each of the eastern, western, southern, and northern regions of Sichuan Province for investigation. Considering that hospitals below the tertiary level lack standardized training qualifications, they were excluded from the study. All eight tertiary hospitals are general hospitals, with a total number of standardized training nurses ranging from 1,000 to 2,000.

### 2.3 Method

#### 2.3.1 Survey contents

① Demographic data: including gender, age, height, education, marital status, relationship with colleagues, monthly income, weekly working hours, etc. ② Chinese Version of the spiritual climate Brief Scale: This instrument, developed by Wu et al. (10), features four Likert 5-point items (ranging from “strongly disagree” to “strongly agree”). The total score ranges from 4 to 20 points, with higher scores indicating a greater perception of spiritual climate. The questionnaire Cronbach’s *α* coefficient of 0.933 demonstrates excellent internal consistency and the content validity index was 0.823. ③ Sonnentag et al. (11) developed the psychological detachment scale in 2007, and Lu et al. (12) translated and revised it to measure the degree to which individuals are detached from work and stop thinking about work affairs during non-working hours. The scale consists of four items, each using a Likert five-point rating system ranging from “strongly disagree” (1 point) to “strongly agree” (5 points), with total scores spanning from 4 to 20 points. Higher scores indicate greater psychological disengagement. The Cronbach’s *α* coefficient of 0.872 ~ 0.873 demonstrates excellent internal consistency and the content validity index was 0.779.

#### 2.3.2 Investigation method

Use Questionnaire Star to conduct an electronic questionnaire survey. Input all the questionnaire contents into the software of Questionnaire Star to make an electronic questionnaire. Each item is set as a mandatory item with unified instructions to explain the filling requirements. Before the survey, with approval from the hospital’s nursing department, a WeChat group was created for standardized training of nurses. Participants were thoroughly briefed on the survey objectives, research scope, and questionnaire requirements before completing the forms independently. To ensure data authenticity, reliability, and sample representativeness, participants were selected from standardized training nurses at tertiary hospitals across different regions. This study will strictly adhere to confidentiality principles and be conducted anonymously, with informed consent options provided to ensure participants’ personal information and privacy are protected. To avoid duplicate submissions, the same mobile phone number or computer IP address can only be used once. Statistical data undergoes dual verification, with rigorous adherence to statistical methods and principles to ensure data authenticity. Researchers collected survey data through the Wenjuanxing platform, excluding non-compliant questionnaires. A total of 729 questionnaires were distributed, and a total of 716 valid questionnaires were collected. With 98.22% valid after collection. The remaining 13 non-compliant questionnaires (including those with <120 s response times or identical answers across all options) were excluded through dual verification by two researchers.

#### 2.3.3 Statistical methods

Data were double-checked, inputted, and analyzed using SPSS 26.0 software. The statistical significance level was set at α = 0.05 for all analyses. Descriptive statistics were applied as follows: Normally distributed measurement data were presented as mean ± standard deviation (mean ± SD), which was selected to accurately reflect the central tendency and dispersion of such continuous data. The count data were described by frequency and percentage, a standard approach to summarize categorical variables by quantifying the proportion of observations in each category. Non-normally distributed measurement data (e.g., spiritual climate and psychological detachment scores) were reported as median M (P25, P75), chosen to avoid skewing by extreme values. Inferential statistics were conducted based on data characteristics: Non-normally distributed measurement data (including spiritual climate and psychological detachment scores) were analyzed using the Mann–Whitney U test or Kruskal–Wallis H test. These nonparametric tests were adopted because they do not require the normality assumption, matching the distributional features of the aforementioned variables. Correlation analysis between variables was performed via Spearman correlation. This method was selected due to the non-normal distribution of key variables (e.g., spiritual climate, psychological detachment), as it quantifies monotonic relationships without relying on the normality assumption. Generalized Linear Models (GLMs) were employed to include and analyze meaningful variables identified in preliminary analyses. For the ordinal and non-normally distributed outcome variables (spiritual climate and psychological detachment scores), a logit link function was used; the models assumed a binomial distribution for binary predictors and an ordinal logistic distribution for ordinal outcome variables, which aligns with the analytical needs of ordinal data by modeling the log-odds of category membership while accommodating non-normality. Before GLM fitting, assumptions were systematically evaluated: The proportional odds assumption (a core requirement for ordinal logistic GLMs) was tested using the Brant test, with results showing no significant violation (all *p* > 0.05), confirming the validity of the logit link function. Multicollinearity among predictor variables was verified via the variance inflation factor (VIF), with all VIF values indicating minimal multicollinearity. Residual analysis was conducted to assess model goodness-of-fit, and residual plots showed no systematic patterns, further supporting the appropriateness of GLM specifications.

## 3 Results

### 3.1 General data

This study was conducted on 716 standardized training nurses in Sichuan Province, including 83 males (11.6%) and 633 females (88.4%); See Table 1 for details.

**Table 1:** Sichuan Provincial standardized training of nurses general data scale (*n* = 716).

Project	Group	Number (*n*)	Percentage (%)
Gender	Man	83	11.6
	Woman	633	88.4
Height	≤155 cm	71	9.9
	155–160 cm	285	39.8
	160–165 cm	216	30.2
	>165 cm	144	20.1
Age	≤20 years old	6	0.8
	21–25 years old	682	95.3
	26–30 years old	28	3.9
Marriage	Unmarried	689	96.2
	Married	27	3.8
Highest education	Junior college	406	56.7
	Undergraduate course	310	43.3
Academic title	Nurse	701	97.9
	Primary nurse	15	2.1
Grades in standardized training	The first year	413	57.7
	The next year	303	42.3
Form of employment	Institute training	138	19.3
	Social training	578	80.7
Number of nurses in your department	≤15	86	12
	16–25	247	34.5
	26–35	113	15.8
	>35	270	37.7
The type of hospital	Special hospital	8	1.1
	General hospital	708	98.9
The level of hospital	Tertiary hospitals	713	99.6
	Secondary hospital	3	0.4
Average weekly hours worked	≤40 h	188	26.3
	40–50 h	455	63.5
	>50 h	73	10.2
Whether you love nursing work	Yes	239	33.4
	Neutral	435	60.8
	No	42	5.9
How well you get along with your colleagues	Good	478	66.8
	Neutral	230	32.1
	Bad	8	1.1
Average monthly income	≤3,000 yuan	422	58.9
	3,001–5,000 yuan	258	36
	>5,000 yuan	36	5.1
Did you work before the training	Yes	84	11.7
	No	632	88.3
Character	Extravert type	195	27.2
	Introvert type	191	26.7
	Commonly	330	46.1

### 3.2 Standardized training nurses perceive the current situation of the spiritual climate

The standardized training nurse spiritual climate score in Sichuan province was 16 (13,16), and the scores of each item are shown in Table 2.

**Table 2:** Scores of spiritual climate items for nurses in standardized training in Sichuan Province (*n* = 716).

Project	Percentile		
	25	50	75
1. The department I work in encourages and supports my opinions and ideas, and my colleagues can listen to and accept them	3.00	4.00	4.00
2. The doctors and nurses respected my opinions and ideas	3.00	4.00	4.00
3. When I share my ideas with colleagues, I feel a sense of belonging and identity	3.00	4.00	4.00
4. In my department, everyone can express their own ideas, we respect each other and understand each other	4.00	4.00	4.00

### 3.3 The current situation of standardized training nurses’ psychological detachment from reality

The standardized training nurse psychological detachment score in Sichuan province was 11 (9,13), and the scores of each item are shown in Table 3.

**Table 3:** Scores of each item of psychological detachment of nurses in standardized training in Sichuan Province (*n* = 716).

Project	Percentile		
	25	50	75
1. After work, I forget about my job	2.00	3.00	4.00
2. After work, I do not think about work at all	2.00	3.00	3.00
3. After work, I will keep myself away from the work	2.00	3.00	4.00
4. After work, I will choose to rest myself even though there are unfinished work	1.00	2.00	3.00

### 3.4 Single factor analysis of spiritual climate and psychological detachment of standardized training nurses

#### 3.4.1 Single factor analysis of spiritual climate for standardized training nurses

The results of one-way factor analysis showed that there were significant differences in the spiritual climate scores of standardized training nurses according to different ages (*p* = 0.027), average weekly working hours (*p* = 0.001), love for nursing work (*p* < 0.001), harmonious relationship with colleagues (*p* < 0.001), average monthly income (*p* < 0.001) and personality (*p* = 0.014). See Table 4 for details.

**Table 4:** Single factor analysis of the spiritual climate of nurses in standardized training in Sichuan Province (*n* = 716).

Project	Number	Score M (P25 ~ P75)	H-number	P-number
Age				
≤20 years old	6	17.5 (15.75 ~ 20)	7.242	0.027
21–25 years old	682	16 (13 ~ 16)		
26–30 years old	28	14 (12.25 ~ 16)		
Average weekly hours worked				
≤40 h	188	16 (14 ~ 17)	15.126	0.001
40–50 h	455	16 (13 ~ 16)		
>50 h	73	14 (12 ~ 16)		
Whether you love nursing work				
Yes	239	16 (16 ~ 20)	108.625	<0.001
Neutral	435	15 (13 ~ 16)		
No	42	13 (11 ~ 15.25)		
How well you get along with your colleagues				
Good	478	16 (15 ~ 18)	106.770	<0.001
Neutral	230	14 (12 ~ 16)		
Bad	8	6 (4 ~ 13)		
Average monthly income				
≤3,000	422	16 (13 ~ 16)	15.245	<0.001
3,001–5,000	258	16 (14 ~ 18)		
>5,000	36	16 (13 ~ 16)		
Character				
Extravert type	195	16 (14 ~ 17)	8.470	0.014
Introvert type	191	16 (14 ~ 17)		
Neutral	330	16 (13 ~ 16)		

#### 3.4.2 The results of one-way factor analysis showed

The results of one-way factor analysis showed that there were significant differences in psychological detachment scores of nurses in standardized training according to different heights (*p* = 0.027) and pre-training work experience (*p* = 0.004). See Table 5 for details.

**Table 5:** Single factor analysis of psychological detachment of nurses in standardized training in Sichuan Province (*n* = 716).

Project	Number of examples	Score	Number	*p*-number
Height				
≤155 cm	71	11 (8 ~ 13)	21411.500^a^	0.027
155–160 cm	285	11 (8 ~ 13)		
160–165 cm	216	11 (9 ~ 13)		
>165 cm	144	12 (10 ~ 14)		
Whether you have worked before the training				
Yes	84	12 (11 ~ 14)	8.397^b^	0.004
No	632	11 (9 ~ 13)		

a Mann–Whitney number.

b Kruskal–Wallis number.

### 3.5 Correlation analysis between spiritual climate and psychological detachment of nurses in standardized training

The spiritual climate of nurses in standardized training was positively correlated with psychological detachment, and the correlation coefficient was 0.081 (*p* = 0.029).

### 3.6 A generalized linear model of spiritual climate and psychological detachment of nurses in standardized training

#### 3.6.1 A generalized linear model of the spiritual climate of nurses in training

Using spiritual climate scores as the dependent variable, this study conducted a generalized linear model analysis based on the following factors: age (reference group: 26–30 years), average weekly working hours (reference group: >50 h), passion for nursing work (reference group: disinterested), harmonious relationships with colleagues (reference group: discordant), average monthly income (>5,000 yuan), and personality type (reference group: normal). The results indicate that key factors influencing the spiritual climate of nurses in standardized training programs include: ① Age: Nurses under 20 years old showed significantly higher spiritual climate scores compared to those aged 26–30 (*β* = 2.643, *p* = 0.034). ② Work attitude: Those passionate about nursing scored significantly higher than those unenthusiastic (*β* = 2.328, *p* < 0.001). ③ Colleague relationships: Harmonious colleagues reported significantly better spiritual climate (*β* = 6.579, p < 0.001). ④ Personality: Introverted nurses scored notably higher than average (*β* = 0.525, *p* = 0.034). Factors such as weekly working hours and monthly income showed no significant impact on spiritual climate (*p* > 0.05). Detailed data are presented in Table 6.

**Table 6:** The generalized linear model of spiritual climate for nurses in standardized training in Sichuan Province.

Project	*β*	Standard error	95% confidence interval		Vald	Valid	P
			Lower limit	Upper limit			
Constant	6.938	1.149	4.685	9.190	36.436	1	<0.001
Age							
≤20 years old	2.643	1.244	0.205	5.081	4.516	1	0.034
21–25 years old	0.835	0.537	−0.217	1.887	2.418	1	0.120
26–30 years old	0^a^						
Average weekly hours worked							
≤40 h	0.511	0.389	−0.252	1.275	1.722	1	0.189
40–50 h	0.100	0.352	−0.590	0.790	0.081	1	0.776
>50 h	0^a^						
Whether you love nursing work							
Yes	2.328	0.501	1.346	3.311	21.573	1	<0.001
Neutral	0.990	0.463	0.082	1.898	4.563	1	0.033
No	0^a^						
How well you get along with your colleagues							
Good	6.579	1.015	4.590	8.567	42.029	1	<0.001
Neutral	5.312	1.009	3.334	7.289	27.719	1	<0.001
Bad	0^a^						
Average monthly income							
≤3,000 yuan	−0.582	0.482	−1.526	0.362	1.460	1	0.227
3,001–5,000 yuan	0.078	0.489	−0.881	1.036	0.025	1	0.874
>5,000 yuan	0^a^						
Character							
Extravert type	0.070	0.249	−0.418	0.557	0.079	1	0.779
Introvert type	0.525	0.248	0.039	1.011	4.483	1	0.034
Neutral	0^a^						

a This parameter is set to zero due to redundancy.

#### 3.6.2 A generalized linear model of psychological detachment in nurses in standardized training

Using psychological detachment scores as the dependent variable and standardized nurse training (SNT) nurses ‘height (with>165 cm as the reference group) and pre-training work experience (with no work experience as the reference group), the results showed that the key factors affecting SNT nurses’ psychological detachment were: ① Height: The psychological detachment score of the 155–160 cm group was significantly lower than that of the>165 cm group (*β* = −1.074, *p* = 0.005). ② Pre-training work experience: Nurses with formal work experience scored significantly higher than those without (*β* = 1.090, *p* = 0.012). The effects of height ≤155 cm and 160–165 cm groups were not statistically significant (*p* > 0.05). The model constant term was 11.794 (*p* < 0.001). Detailed results are shown in Table 7.

**Table 7:** The generalized linear model of psychological detachment of nurses in standardized training in Sichuan Province.

Project	*β*	Standard error	95% confidence interval		Vald	Valid	*p*
			Lower limit	Upper limit			
Constant	11.794	0.318	11.172	12.416	1380.879	1	<0.001
Height							
≤155 cm	−0.964	0.543	−2.029	0.101	3.146	1	0.076
155–160 cm	−1.074	0.383	−1.825	−0.324	7.868	1	0.005
160–165 cm	−0.760	0.403	−1.551	0.030	3.554	1	0.059
>165 cm	0^a^						
Did you work before the training							
Yes	1.090	0.43600	0.235	1.944	6.249	1	0.012
No	0^a^						

a This parameter is set to zero due to redundancy.

## 4 Discussion

### 4.1 Current situation and influencing factors of spiritual climate

#### 4.1.1 Current situation of the spiritual climate of standardized training nurses

This study showed that the spiritual climate of nurses in standardized training in the eight hospitals was at a medium level (median 16 points), which was consistent with the previous research of the research group (13), reflecting the optimization space of the department’s humanistic environment. The “Department Encourages Feedback Adoption” and “Colleague Respect & Belonging” items in the spiritual climate evaluation showed higher scores, indicating that medical institutions in the eight hospitals have established a foundation in humanistic management. However, the relatively low score for “Department Respect for Individual Expression” may be related to the limited voice rights of standardized training nurses within hierarchical systems (14).

#### 4.1.2 Influencing factors of spiritual climate perception

Key factors influencing nurses’ in standardized training perception of spiritual climate include: ① Age: The score for the ≤20-year-old group was significantly higher than that of the 26-30-year-old group (*β* = 2.643, *p* = 0.034), which may be related to new nurses’ strong professional adaptability and higher expectations for work environments, while senior nurses face greater gaps between reality and ideals (15). ② Work Attitude: Nurses with stronger passion showed significantly higher spiritual climate scores (*β* = 2.328, *p* < 0.001), indicating that professional identity is the core driving force for creating a positive departmental atmosphere. ③ Colleague Relationships: Those with harmonious relationships demonstrated significantly higher spiritual climate scores compared to those with discordant relationships (*β* = 6.579, *p* < 0.001), confirming that harmonious interpersonal interactions form the foundation of spiritual climate. ④ Individual Traits: Introverted nurses scored higher than those with average personalities (β = 0.525, *p* = 0.034), which may relate to introverts’ heightened sensitivity to supportive environments. It is worth noting that working hours and income did not significantly affect the spiritual climate, suggesting that non-material factors (such as respect and sense of belonging) play a more important role in shaping the departmental atmosphere.

### 4.2 The status quo and influencing factors of psychological detachment

This study demonstrates that mid-level psychological detachment (median 11 points) exists among nurses who are in standardized training in these eight hospitals, consistent with the findings of Lu Dezhi et al. (11) and showing significant correlations. Height and detachment capacity: Nurses taller than 165 cm exhibited significantly better psychological detachment than those between 155 and 160 cm (*β* = −1.074, *p* = 0.005). However, this correlation should be interpreted cautiously, as we did not collect physiological (e.g., physical fatigue threshold) or ergonomic (e.g., work posture adaptation) data to verify the potential mediating mechanisms (e.g., workload tolerance). Work experience value: Nurses with prior formal work experience demonstrated stronger psychological detachment capabilities (*β* = 1.090, *p* = 0.012), indicating that previous professional experience may enhance self-regulation skills in stress management and work boundary adaptation.

### 4.3 The weak correlation between spiritual climate and psychological detachment

(*r* = 0.081, *p* = 0.029) is statistically significant but with a limited effect size. This finding validates the core tenet of the Effort-Recovery Theory (16). A high-quality spiritual climate enhances nurses’ perceived organizational support and reduces emotional workload, thereby promoting psychological resource recovery (17, 18). When nurses feel respected for their professional expertise in their departments, their sense of job control increases, facilitating cognitive detachment during non-work hours. Harmonious collegial relationships also help buffer work-related stress from eroding psychological boundaries (19–22). The low effect size suggests: ① The enhancement of psychological detachment through improved departmental spiritual climate may be influenced by mediating/ moderating variables (e.g., workload, individual resilience); ② As the core component of recovery processes, psychological detachment demonstrates strong independence, requiring targeted interventions (e.g., mindfulness training) rather than relying solely on environmental optimization.

### 4.4 Practical insights

① Departmental Management Level: Focus on building a respectful and inclusive team culture (e.g., establishing feedback mechanisms), strengthening peer support networks, with particular attention to the integration of nurses with low professional identity (5.9%). ② Training System Design: Add stress management and work-life balance courses for pre-service trainees without work experience (88.3%), while exploring differentiated shift scheduling strategies based on height-related physical workload.

## 5 Conclusion

This study reveals that the spiritual climate (median: 16 points) and psychological disengagement (median: 11 points) among standardized training nurses in the eight hospitals are at moderate levels. Spiritual climate is positively influenced by age ≤20 (*β* = 2.643), professional passion (*β* = 2.328), harmonious colleague relationships (*β* = 6.579), and introverted personality (*β* = 0.525). Psychological disengagement shows significant correlations with height (>165 cm group shows better results) and pre-training work experience (*β* = 1.090). Although there exists a weak positive correlation between spiritual climate and psychological disengagement (*r* = 0.081, *p* = 0.029), the effect is limited, indicating the need for targeted interventions. Recommendations: ① Department management should strengthen humanistic atmosphere to enhance the sense of belonging among nurses with low professional identity; ② Training systems should incorporate stress management courses to improve recovery capabilities for nurses without work experience. The study’s limitations stem from its cross-sectional design, requiring future longitudinal verification of causal mechanisms.

## 6 Limitations and future directions

Due to the limitation of non-random sampling (convenience sampling), this study may still have the following selection bias, which needs to be objectively discussed: This study was conducted exclusively in tertiary hospitals within Sichuan Province, resulting in geographical limitations in sample representativeness. Besides The cross-sectional design cannot establish causal relationships and requires longitudinal follow-up verification. Workload quantification indicators (e.g., number of patients cared for) were not included, necessitating future integration of objective data to deepen mechanistic analysis. We acknowledge that factors such as department assignment (e.g., intensive care units vs. general wards with different workloads), workload demands (e.g., daily patient care hours), and shift patterns (e.g., night shift frequency) may interfere with the observed correlation. Due to the cross-sectional nature of this study, we could not control these variables through longitudinal tracking, which is a limitation to be addressed in future research. Subsequent studies will integrate physiological measurement tools (e.g., heart rate variability monitors) and collect detailed work context data to explore whether height indirectly affects psychological detachment through mediating factors (e.g., physical stress resistance) and control for confounders via multivariate regression models.

## Data Availability

The original contributions presented in the study are included in the article/supplementary material, further inquiries can be directed to the corresponding author.
